# Analysis of the IgE epitope profile of soybean allergen Gly m 4

**DOI:** 10.1186/2045-7022-4-S2-P42

**Published:** 2014-03-17

**Authors:** Felix Husslik, Diana Mittag, Christian Seutter von Loetzen, Maximilian Hartl, Lothar Vogel, Barbara Ballmer-Weber, Jörg Kleine-Tebbe, Regina Treudler, Jan-Christoph Simon, Kay-Martin Hanschmann, Andreas Reuter, Michaela Gubesch, Paul Rösch, Stefan Vieths, Thomas Holzhauser, Dirk Schiller

**Affiliations:** 1 Paul-Ehrlich-Institut, Division of Allergology, Langen, Germany; 2 Australian Red Cross Blood Service, Research & Development Division, Melbourne, Australia; 3 University of Bayreuth, Department of Biopolymers, Bayreuth, Germany; 4 University Hospital Zurich, Allergy Unit, Department of Dermatology, Zurich, Switzerland; 5 Allergy and Asthma Center Westend, Praxis Hanf, Ackermann & Kleine-Tebbe, Berlin, Germany; 6 University of Leipzig, Department of Dermatology and Allergology, Leipzig, Germany; 7 Paul-Ehrlich-Institut, Department of Biostatistics, Langen, Germany

## Background

Individuals with birch pollinosis may show allergic reactions after consumption of soybean-containing food. This is caused by cross-reaction of IgE directed against the major birch pollen allergen Bet v 1 with its homologue Gly m 4 from soybean. Birch pollen allergic subjects without soybean allergy often present IgE binding to Gly m 4.

## Objective

To identify IgE epitopes of Gly m 4 in birch pollen-allergic subjects with and without allergy to soybean.

## Methods

Sera from 33 birch pollen-allergic patients with and without clinical reactivity to soy as determined by oral food challenge or convincing history of soy allergy were included in the study. Specific IgE against Bet v 1 and Gly m 4 was determined by ImmunoCAP. IgE binding of the sera to rGly m 4 and soy extract was tested in western blot analysis. To predict putative IgE epitopes, 20 IgE-binding phage-displayed peptide mimics and heptamers representing the total Gly m 4 amino acid sequence were bioinformatically mapped onto the molecular surface of Gly m 4. To verify predicted epitopes, Gly m 4 variants with substituted epitope residues were expressed in and purified from *E. coli*. Secondary and tertiary structures were assessed by CD and NMR spectroscopy. IgE binding of variants was analyzed by competitive immunoblotting and inhibition ELISA.

## Results

Gly m 4- and Bet v 1-specific IgE levels ranged from 0 to >100 kU_A_/L in sera of both patient groups. CAP values were above 3.5 kU_A_/L in 18 sera (86%) of patients with clinical soy allergy and in 7 sera (58%) of patients sensitized, but without clinical reactivity to soy, respectively. Intensity of IgE binding in western blot corresponded to ImmunoCAP analysis. Bioinformatic mapping resulted in four distinct epitopes on Gly m 4 surface. Several amino acid residues were chosen for generation of substitutional variants. Purified rGly m 4 variants showed a typical Bet v 1-like structure according to CD spectra and 1D-^1^H-NMR spectroscopy. IgE binding to several substitutional variants was reduced, indicating a multiple number of epitopes on Gly m 4 surface.

## Conclusion

No significant differences in Gly m 4-specific IgE binding could be observed between birch pollen-allergic patients with and without clinical soy allergy in ImmunoCAP and immunoblot analysis. However residual IgE reactivity persists in substitutional rGly m 4 variants, indicating the presence of further IgE epitopes not yet identified.

